# Effects of Intrauterine Infusion of a Chitosan Solution on Recovery and Subsequent Reproductive Performance of Early Postpartum Dairy Cows with Endometritis: A Pilot Field Trial

**DOI:** 10.3390/ani11010197

**Published:** 2021-01-15

**Authors:** Hiroaki Okawa, Missaka M.P. Wijayagunawardane, Peter L.A.M. Vos, Osamu Yamato, Masayasu Taniguchi, Mitsuhiro Takagi

**Affiliations:** 1 United Graduate School of Veterinary Science, Yamaguchi University, Yamaguchi 753-8515, Japan; k2414076@kadai.jp (H.O.); osam@vet.kagoshima-u.ac.jp (O.Y.); masa0810@yamaguchi-u.ac.jp (M.T.); 2 Fukuoka Prefecture Dairy Cooperative Association, Fukuoka 819-0373, Japan; 3 Guardian Co. Ltd., Kagoshima 890-0033, Japan; 4 Department of Animal Science, University of Peradeniya, Peradeniya 20400, Sri Lanka; missakaw@pdn.ac.lk; 5 Department Population Health Sciences, Farm Animal Health, Section Reproduction, Utrecht University, Yalelaan 7, 3584 CL Utrecht, The Netherlands; P.L.A.M.Vos@uu.nl; 6 Joint Faculty of Veterinary Medicine, Kagoshima University, Kagoshima 890-0062, Japan; 7 Joint Faculty of Veterinary Medicine, Yamaguchi University, Yamaguchi 753-8515, Japan

**Keywords:** chitosan, dairy cow, endometritis, polymorphonuclear leukocytes, treatment

## Abstract

**Simple Summary:**

Endometritis is one of the most common disorders during the postpartum period in dairy cows. We investigated the efficacy of intrauterine infusion of a chitosan solution in uterine recovery in early postpartum dairy cows with or without endometritis, and their subsequent reproductive performance. We found that, compared to the absence of treatment, chitosan solution treatment during the early postpartum period (approximately 3 weeks after parturition) accelerated uterine recovery after parturition of dairy cows. These findings suggest that the administration of chitosan solution once in the early postpartum period may have antimicrobial effects on the uterus. We suggest that chitosan solution is a potential therapeutic candidate for endometritis that may replace prostaglandin F_2α_ or antibiotic treatments_._

**Abstract:**

This study investigated the efficacy of intrauterine infusion of a chitosan solution (CHT) on uterine recovery in early postpartum dairy cows with or without endometritis, and their subsequent reproductive performance. In Experiment 1, cows with endometritis at 3 weeks postpartum were administered CHT (n = 5) and prostaglandin F_2α_ (PGF_2α_) (n = 4). Untreated cows (n = 7) served as the control group. In Experiment 2, 18 cows with a normally recovered uterus at the fresh cow check (mean, 35 days postpartum) were assigned to the CHT (n = 10) and control (n = 8) groups, and intrauterine infusion was conducted in the CHT group. Overall, in Experiment 1, the percentage of polymorphonuclear leukocytes significantly declined in the CHT group (32.3 ± 10.2 to 5.5 ± 2.4, *p* < 0.05) from week 3 to week 5, but no decline occurred in the PGF_2α_ and control groups. In Experiment 2, the CHT and control groups showed no significant differences in reproductive parameters, suggesting the absence of adverse effects of CHT on fertility. These results suggest that intrauterine infusion of CHT in the early postpartum period effectively accelerates uterine recovery from endometritis and might be a suitable replacement for PGF_2α_ administration.

## 1. Introduction

Endometritis is one of the most common disorders during the postpartum period in dairy cows. Clinical endometritis is defined as the occurrence of purulent or mucopurulent vaginal discharge detected more than 3 weeks postpartum [1]. We previously evaluated the efficiency of a grading system based on the vaginal discharge score (VDS) using vaginoscopy under field conditions [2]. The results reveal that early treatment with prostaglandin F_2α_ (PGF_2α_) (within 40 days postpartum) might be effective in improving the reproductive performance of cows with clinical endometritis. However, the effect of PGF_2α_ treatment on early postpartum endometritis is controversial since the treatment effect varies according to the presence or absence of a functional corpus luteum (CL) [3]. Although antimicrobial treatment is beneficial for cows with endometritis, problems such as the emergence of antimicrobial-resistant bacteria are present [4]. Moreover, in Japan, the use of commercial drugs approved for the intrauterine treatment of endometritis is limited by regulations related to milk withdrawal periods. Hence, farmers prefer avoiding the use of antimicrobial agents in high-producing dairy cows during the early postpartum period. Therefore, effective and safe treatments that exclude antimicrobial agents and do not have withdrawal periods are needed.

Chitin is a polysaccharide that occurs naturally in the skeletons of crustaceans and the cell walls of fungi, and chitosan is commercially produced by the deacetylation of chitin. Chitosan has been widely used in many industries, including food, agriculture, and medical sciences, and it has no drawbacks related to the withdrawal period [5,6,7]. Indeed, chitosan is used as a wound-healing accelerator to induce a migration response from polymorphonuclear (PMN) cells and to enhance local biological defense mechanisms in veterinary practice [5,8,9]. Suzuki et al. [9] reported that intrauterine infusion of chitin solution induced estrus and that it possibly has a therapeutic effect on endometritis in Japanese Black cows. In addition, intrauterine administration of chitosan microparticles at the very early postpartum period, such as within 1 week after calving, has been reported to reduce the incidence of metritis [6,7]. Chitosan has shown broad-spectrum antimicrobial activity in both in vivo and in vitro animal models; thus, it has the potential to be used as an alternative antimicrobial agent to control pathogens and to enhance host defense systems in the uterine environment [6,7]. However, the effectiveness of an intrauterine infusion of a chitosan solution based on the number of PMN cells in the uterus remains unclear under field conditions, not only in cows with endometritis at 14–21 days postpartum but also in normally recovered cows without endometritis at 30–40 days postpartum during the fresh cow check. We hypothesized that for cows with endometritis, the intrauterine infusion of a chitosan solution would have beneficial antimicrobial and host defense effects in the uterine environment and would accelerate the cow’s ability for spontaneous uterine recovery, thus having a positive effect on the cow’s subsequent reproductive performance. In contrast, in normally recovered postpartum cows without endometritis, intrauterine infusion of a chitosan solution would have no negative/adverse effects on their uterine environment or fertility. The administration of a chitosan solution to prevent endometritis, as a replacement for PGF_2α_, may also have a positive effect on the subsequent reproductive performance of dairy herds.

Therefore, the objective of this field study was to (1) evaluate the effect of intrauterine infusion of a chitosan solution in cows at 3 weeks postpartum diagnosed with clinical endometritis and to compare it with the effect of intramuscular administration of PGF_2α_ on uterine recovery and subsequent reproductive efficacies, and (2) evaluate the effect of intrauterine infusion of a chitosan solution at the fresh cow check on subsequent reproductive performance in normally recovered postpartum cows without endometritis.

## 2. Materials and Methods

### 2.1. Experiment 1: Comparison of the Effects of Intrauterine Infusion of a Chitosan Solution Versus Intramuscular Administration of PGF_2α_ on Uterine Recovery in Cows with Endometritis

This experiment was conducted at a dairy farm in Fukuoka Prefecture, Japan. All cows were housed in tie stalls with rubber mattresses, fed twice daily with a total mixed ration, and milked twice daily with an average milk production of 9579 kg/cow/lactation. Cows that had calved from October 2017 to February 2018 were used in this study. Cows that developed peripartum diseases, such as milk fever, metritis, displaced abomasum, or mastitis, after parturition were excluded from the study. The voluntary waiting period in the herd was set at 60 days postpartum. Cows were bred via artificial insemination (AI) performed by local technicians. Pregnancy was diagnosed using transrectal palpation 50 days after the previous insemination. For cows that were not inseminated within 90 days postpartum or that had not become pregnant at the time of pregnancy diagnosis, additional investigation and treatment were performed, including a timed AI program using a combined injection of PGF_2α_ and estradiol. Briefly, 2 mg of estradiol benzoate (Ovahormon^®^; ASUKA Animal Health Co., Ltd., Tokyo, Japan) was administered intramuscularly and a controlled internal drug-releasing device (CIDR; CIDR^®^ 1900; Zoetis Japan Co., Ltd., Tokyo, Japan) was inserted on day 0; intramuscular administration of 500 µg of cloprostenol (Estrumate^®^; Intervet K.K, Tokyo, Japan) was performed on day 9 or 10, along with the removal of the CIDR and 1 mg of estradiol benzoate was administered on day 10 or 11 followed by AI on day 11 or 12.

In this study, 24 cows underwent examinations for ovarian and uterine conditions at approximately week 2 (W2; 14.6 ± 0.3 days), week 3 (W3; 21.5 ± 0.3 days), and week 5 (W5; 35.8 ± 0.4 days) after parturition. Uterine conditions were evaluated and classified. We also established a uterine condition score (UCS) system at W2, W3, and W5 with reference to our previous report [2]: UCS1 = the uterus was palpable entirely, with symmetry of the uterine horns and confirmed normal uterine conditions; UCS2 = the uterus was palpable entirely, but with asymmetry of the uterine horns and incomplete involution; and UCS3 = the uterus was not palpable entirely and was outside the pelvic cavity. Simultaneously, the ovary was palpated, and the presence of palpable ovarian structures was recorded.

The body condition score (BCS) was evaluated on a 5-point scale with increments of 0.25 at W2, W3, and W5, as described by [10]. The VDS was determined at W2, W3, and W5 using a Metricheck device (Simcro Tech Ltd., Hamilton, New Zealand) that comprised a stainless-steel rod and a silicon cup. The VDS assessment method was similar to the method described by [11] with slight modifications as follows: VDS1 = clear or translucent mucus; VDS2 = mucus containing flecks of white pus or off-white purulent material; VDS3 = discharge containing <50% purulent material; and VDS4 = discharge containing >50% purulent material.

Endometrial samples were collected using a cytobrush (Metribrush; Fujihira Industry Co., Ltd., Tokyo, Japan) as described previously [12,13] at W3 and W5. The vulva was washed, wiped using a paper towel, and the instrument was inserted through the vagina and cervix into the uterine cavity. Endometrial samples were collected by rotating the brush while in contact with the endometrium. Cytology slides were prepared by rolling the brush containing the tissue samples onto two clean microscopic slides. The slides were then fixed, stained with Diff Quik Solution (Sysmex Corp., Kobe, Japan), washed, and air-dried. Cytological assessment was performed to determine the percentage of polymorphonuclear leukocytes (PMNL%) by counting a minimum of 200 nucleated cells, as previously described [14].

Twenty-four cows were randomly assigned to three groups of eight cows each. The CHT group was treated with an intrauterine infusion of 50 mL chitosan solution containing 0.8% chitosan (pH: 4.0–4.6) in a low concentration of brewed vinegar (0.5%) (CHITOSAID^®^C; Meiji Seika Pharma Co., Ltd., Tokyo, Japan). The dose of CHT used in the present study was similar to that described in previous reports [9,15]. The PGF_2α_ group was treated with intramuscular PGF_2α_ (25 mg of dinoprost, Pronalgon^®^F; Zoetis Japan Co., Ltd., Tokyo, Japan). The control group did not receive any treatment during the experimental period and served as the control. Since this is a clinical pilot study on the farm, and a control group was required, it was ethical to use the minimum required number of examined animals. The treatments and examinations were simultaneously conducted at W3.

In this study, the diagnosis of endometritis was defined as a PMNL% ≥ 8 according to previously accepted criteria [16]. Three cows in the CHT group, four in the PGF_2α_ group, and one in the control group were later diagnosed as not having endometritis (PMNL% < 8); hence, they were excluded from this study. Therefore, the final sample sizes were five, four, and seven cows each in the CHT, PGF_2α_, and control groups, respectively. The cows with no endometritis at W3 did not receive any further treatment or examination at W5. The reproductive performances compared among the three groups included the calving to first AI (FAI) interval, successful conception after FAI, days open, and number of AIs to become pregnant and days open.

### 2.2. Experiment 2: Evaluation of the Effects of Intrauterine Infusion of CHT on Reproductive Efficacy Based on the Number of PMN Cells in Normally Recovered Postpartum Cows without Endometritis

This experiment was conducted at seven dairy farms in Fukuoka Prefecture, Japan. All cows were housed in tie stalls with rubber mattresses, fed twice daily with a total mixed ration, and milked twice daily with an average milk production of 8981–11,453 kg/cow/lactation. Cows that had calved from October 2017 to July 2019 were used in this study. The methods of selection and postpartum treatments of the examined cows were identical to those in Experiment 1. In this experiment, cows classified into the UCS1 category based on rectal palpation performed as a postpartum fresh cow check between 28 and 40 days in milk (DIM) were randomly assigned to the CHT and control groups. Additionally, endometrial samples were collected using a cytobrush as described above for subsequent cytological confirmation of the diagnosis as normally recovered without endometritis. Thereafter, the CHT group was treated with an intrauterine infusion of 50 mL chitosan solution as described above. After the administration of CHT, the reproductive management of all cows was conducted as described above.

### 2.3. Statistical Analysis

All statistical analyses were performed using the BellCurve for Excel software program (Social Survey Research Information Co., Ltd., Tokyo, Japan). Results are expressed as the mean ± standard error of the mean (SEM) or as a percentage (%). The results for the parameters BCS, UCS, VDS, PMNL%, and FAI data obtained from the three groups at W2, W3, and W5 were analyzed using repeated measures one-way analyses of variance. In addition, differences between PMNL% at W3 and W5 were analyzed using a paired *t*-test. One-way ANOVA was used for the comparisons between the groups (control, PGF_2α_, and CHT). Successful conception after FAI was compared using Fisher’s exact probability test. Post hoc power analysis was performed in each experiment to confirm the power in the sample size of this study. *P* < 0.05 was considered statistically significant.

## 3. Results

### 3.1. Experiment 1

Cow parity ranged from 1 to 4, with an average parity of 1.9 ± 0.3. Parity had no significant effect in any of the three groups. Table 1 shows the postpartum changes in the BCS, UCS, VDS, and PMNL% at W2, W3, and W5. Although all cows showed a slight decrease in the BCS, it had no significant effect. The UCS and VDS also decreased on each passing postpartum day with significance within each group, but without significant differences among the three groups. In the present study, 75% (12/16) of the examined cows were diagnosed with clinical endometritis based on the VDS score results before the CHT infusion at W3. The PMNL% was not significantly different among the three groups at W3 and W5. However, the PMNL% showed a significant decrease from W3 to W5 in the CHT group (*p* < 0.05), but not in the PGF_2α_ and control groups. Table 2 shows the reproductive performance of the cows. No significant differences were observed among all parameters. Additionally, the parturition of the five cows, as well as the calves, in the CHT group was confirmed to be normal. When aiming to detect the effect size of partial η^2^ = 0.5, the power in this number of cases was 82.1%.

### 3.2. Experiment 2

The PMNL% of the cows in both the CHT (1.5 ± 0.8) and control (2.4 ± 0.6) groups at the postpartum fresh cow check was less than 8% and was not significantly different. Thus, taken together with the diagnosis performed using the UCS system at the fresh cow check, these results confirm that the postpartum cows used in Experiment 2 had recovered normally. Table 3 shows the parity, DIM of sampling, FAI interval, days open, and number of AIs before pregnancy in both the CHT (n = 10) and control (n = 8) groups. No significant differences among parameters were observed between the two groups. When aiming to detect the effect size of partial η^2^ = 0.5, the power in the number of cases was 97.8%.

## 4. Discussion

We previously examined the effects of diagnosis and treatment of clinical endometritis based on the VDS grading system in postpartum Holstein cows, and the results indicate that cows with endometritis treated with PGF_2α_ (VDS > 2) recovered and regained fertility more quickly than did untreated cows, and their recovery and fertility were comparable to those of cows without endometritis (VDS = 0). However, cows diagnosed with mild endometritis (VDS = 1) that did not receive treatment showed lower reproductive efficiency; thus, early postpartum treatment around the day of the fresh cow check (approximately 29–40 days postpartum) may have had positive effects on the management of dairy herds [2]. Based on our previous results, we speculated that CHT may be a potential candidate that can be used instead of PGF_2α_ and antimicrobial agents in early postpartum cows. In Experiment 1, we attempted to validate the efficacy of CHT and to compare it to that of PGF_2α_ under field conditions in early postpartum cows with endometritis. Our findings demonstrate that CHT treatment during the early postpartum period (3W) accelerated uterine recovery after parturition, as evaluated using the PMNL%, compared to the absence of treatment.

In the present study, the cows treated with PGF_2α_ at an early stage (3W) showed no significant improvement in uterine condition compared to cows treated with CHT. LeBlanc et al. [3] reported that PGF_2α_ treatment of cows with endometritis within W4 had no benefit on their reproductive performance, and that the use of PGF_2α_ for cows with endometritis with no corpus luteum was associated with a reduction in the pregnancy rate, which might be supported by a later conducted meta-analysis [17,18]. Therefore, the above report may support our data, highlighting the reduced effectiveness of PGF_2α_ administration. PGF_2α_ is necessary for the process of spontaneous uterine involution [19,20,21]. Kawashima et al. [11] reported that the blood concentration of the PGF_2α_ metabolite 13,14-dihydro-15-keto-PGF_2α_ (PGFM) tended to be higher in cows with delayed spontaneous uterine involution and lower in cows with endometritis than in healthy cows during W3. The cows with normal uterine involution showed a negative correlation between the blood PGFM level and the time to complete uterine involution. This may reflect that the inflammatory status of cows with normal uterine recovery is both reduced and weak during the early postpartum period. Indeed, a recent study reported that a weekly PGF_2α_ administration protocol (three intramuscular injections at W1, W2, and W3, which may affect the reduced and weak inflammatory status) was effective in reducing endometritis and improving the subsequent reproductive performance of dairy cows [22]. Taken together with our findings, these findings reveal that the administration of CHT once at the early postpartum period (W3) may have antimicrobial effects on the uterus for at least 2 weeks. Therefore, CHT is a potential therapeutic candidate for endometritis. However, further studies are needed to evaluate the mechanism underlying the effectiveness of CHT administration on the resumption of uterine function during the early postpartum period, especially the antibacterial effects of CHT in vitro. Furthermore, a small amount of brewed vinegar was added to the chitosan preparation used in the present study in order to melt it to make it water-soluble. In the future, it may be necessary to examine the possibility that this added brewed vinegar may have affected its therapeutic effects as well as the intrauterine pH and osmotic pressure.

Previously, Suzuki et al. [9] evaluated the therapeutic effects of intrauterine administration of a chitin suspension in both endometritis treatment and estrus induction in cows and reported that the pus associated with endometritis in cattle disappeared after the intrauterine infusion of the chitin suspension. Kohiruimaki et al. [15] also reported that intrauterine administration of a chitosan solution for postpartum anestrus cows had a positive effect on reproductive performance (higher pregnancy rate) when used in combination with a controlled internal drug release (CIDR) short program (i.e., the CIDR and timed AI program initiated 7 days after the infusion of a chitosan solution). However, the intrauterine administration of chitin or chitosan solutions to cows in previous studies was not performed at the early postpartum periods, and thus, the effect of a chitosan solution on uterine recovery from endometritis in the early postpartum period around the time of the fresh cow check had not yet been clearly investigated. In this study, a more significant decrease in the PMNL% from W3 to W5 was observed in the CHT group than in the control and PGF_2α_ groups. Therefore, our results indicate that CHT administration at W3 had a beneficial effect on the recovery from endometritis during the early postpartum period in dairy cows. Indeed, a previous study reported that intrauterine administration of 8 g/40 mL chitosan microparticle solution to prevent metritis in dairy cows for 5 days after parturition had no effect on the clinical responses (rectal temperature and plasma haptoglobin, plasma non-esterified fatty acid, or β-hydroxybutyrate acid concentrations), but the treated cows showed a decreased incidence of metritis at 7 days postpartum [6]. Additionally, chitosan microparticles at a 0.2% concentration showed antimicrobial activity against pathogenic *Escherichia coli* in the uterine fluid in vivo and had a beneficial effect on recovery from metritis [7,23]. Thus, the intrauterine infusion of a chitosan solution is speculated to stimulate the local immune system by inducing leucocyte migration, which may be the reason for the strong antimicrobial activity shown [5,7,8]. Therefore, in the present study, although the cows in the early postpartum period up to 21 days are in the process of spontaneous uterine recovery from parturition, we speculated that the intrauterine infusion of a chitosan solution may accelerate uterine recovery by providing antimicrobial activity and enhancing the local biological intrauterine defense mechanisms. On the other hand, as shown in Table 2, no significant difference was confirmed in the effect of intrauterine administration of CHT on reproductive performance in the pilot study. However, the effect size on days open shows a certain size of 0.184, and it is expected that significance detection will be possible by securing the number of cases in the future.

Owing to its low toxicity, CHT is widely used in many areas, including the food, pharmaceutical, agriculture, and cosmetic industries, and is generally categorized as being safe [7]. However, to our knowledge, no study has evaluated the clinical response after intrauterine infusion of CHT in early postpartum cows with a normally recovered uterus. Our results from Experiment 2 indicate that all parameters regarding reproductive efficacy were similar between the CHT and control groups. Moreover, we confirmed that the parturition process of cows and their newborn calves was clinically normal. Therefore, CHT administration did not have any adverse effect on postpartum cows with a normally recovered uterus; on the other hand, it did not seem to have any beneficial effect of reproductive efficacy, such as increased fertility. In light of the results of Experiments 1 and 2, we suggest that the intrauterine infusion of CHT is performed during the fresh cow check of postpartum dairy cows to prevent reproductive loss due to subclinical endometritis. We have also previously reported the factors associated with impaired reproductive performance in cows with calving abnormality, retained placenta, etc. [24]. These findings highlight the need for further clinical trials employing the intrauterine infusion of a chitosan solution in cows with an increased risk of endometritis as well as the evaluation of parameters associated with the clinical and biochemical findings, which may be vital in determining the effect of a chitosan solution on reproductive performance.

## 5. Conclusions

In conclusion, as a pilot study, we demonstrated the possibility that intrauterine infusion of a chitosan solution could accelerate uterine recovery from endometritis during the early postpartum period. It is essential to confirm not only the need for further research on a large number of cows employing the intrauterine infusion of CHT, but also the antibacterial effects of the chitosan solution used in the present study, especially in in vitro systems.

## Figures and Tables

**Table 1:** Changes in the BCS, UCS, VDS, and PMNL% at W2, W3, and W5.

Group	Parameter	W2	W3	W5	ES	*p*-Value
Control	BCS	3.1 ± 0.1	3.0 ± 0.1	2.9 ± 0.1	0.250	0.178
	UCS	2.9 ± 0.4	1.7 ± 1.0	1.1 ± 0.1	0.762	<0.001
	VDS	2.3 ± 0.2	2.0 ± 0.4	0.6 ± 0.2	0.681	0.001
	PMNL%	-	21.2 ± 3.8	14.1 ± 6.8	0.162	0.323
PGF2a	BCS	3.1 ± 0.1	2.9 ± 0.1	2.9 ± 0.1	0.542	0.096
	UCS	2.8 ± 0.2	2.0 ± 0.4	1.3 ± 0.2	0.844	0.004
	VDS	3.0 ± 0.2	1.8 ± 0.2	0.8 ± 0.2	0.981	0.019
	PMNL%	-	27.1 ± 12.8	10.6 ± 4.1	0.334	0.307
CHT	BCS	3.3 ± 0.1	3.2 ± 0.1	2.8 ± 0.1	0.752	0.004
	UCS	2.8 ± 0.2	1.6 ± 0.2	1.0 ± 0.0	0.840	0.001
	VDS	2.6 ± 0.2	2.2 ± 0.3	1.4 ± 0.4	0.509	0.058
	PMNL%	-	32.3 ± 10.2 ^a^	5.5 ± 2.4 ^b^	0.681	0.043

^a, b^: Significant difference (*p* < 0.05) between the columns; BCS, body condition score; UCS, uterine condition score; VDS, vaginal discharge score; PMNL%, percentage of polymorphonuclear leukocytes; W2, week 2; W3, week 3; W5, week 5; Control, no treatment; PGF2a, intramuscular administration of PGF2a; CHT, intrauterine infusion of 0.8% chitosan in a low concentration of brewed vinegar; ES, effect size: partial η^2^.

**Table 2:** Reproductive performance and percentage of cows in all three groups.

	Control (n = 7)	PGF2α (n = 4)	CHT (n = 5)	ES	*p*-Value
CL palpable at treatment	42.9% (3/7)	50.0% (2/4)	60.0% (3/5)	0.147	0.999
Calving to FAI interval	84.4 ± 10.3	94.3 ± 21.8	76.2 ± 8.0	0.060	0.670
Conception after FAI	0.0% (0/7)	25.0% (1/4)	20.0% (1/5)	0.338	0.300
Days open	186.5 ± 20.4	211.5 ± 59.3	127.0 ± 27.1	0.184	0.296
Number of AIs	4.0 ± 0.9	3.25 ± 1.0	2.25 ± 0.6	0.137	0.414

Control, no treatment; PGF2α, intramuscular administration of PGF2α; CHT, intrauterine infusion of 0.8% chitosan in a low concentration of brewed vinegar; CL, corpus luteum; FAI, first artificial insemination; ES, effect size: partial η^2^ or Cramer’s V (for categorical data).

**Table 3:** Reproductive performance of cows whose uterus recovered normally at the time of the fresh cow check with or without the administration of a chitosan solution.

	CHT (n = 10)	Control (n = 8)	ES	*p*-Value
Parity	3.2 ± 0.9	2.9 ± 0.6	0.005	0.784
DIM of sampling	35.0 ± 1.6	34.4 ± 1.1	0.006	0.768
FAI interval	96.7 ± 7.8	90.5 ± 8.1	0.018	0.593
Days open	142.6 ± 15.6	135.9 ± 13.9	0.006	0.758
Number of AIs	1.8 ± 0.2	2.1 ± 0.4	0.031	0.482

CHT, with chitosan solution administration; control, without chitosan solution administration; DIM, days in milk; FAI, first artificial insemination postpartum; ES, effect size: partial η^2^.

## Data Availability

Not applicable.
